# Bilateral anterior sternothoracotomy (clamshell incision): a suitable alternative for bilateral lung sarcoma metastasis in children

**DOI:** 10.1186/1477-7819-12-233

**Published:** 2014-07-27

**Authors:** Olivier Abbo, Ramona Guatta, Kalitha Pinnagoda, Jean-Marc Joseph

**Affiliations:** 1 Service de Chirurgie Viscérale Pédiatrique, Hôpital des Enfants de Toulouse, CHU, Toulouse, France; 2 Service de Chirurgie Pédiatrique, CHUV, 1011 Lausanne, Switzerland

## Abstract

**Background:**

The aim of our study was to assess the postoperative course of bilateral anterior sternothoracotomy (BAT) in children with sarcoma metastases, in a curative care perspective.

**Methods:**

We reviewed the records of seven patients younger than 18 years old, who underwent surgical procedures for sarcoma metastasis to the lung between 2000 and 2012. We compared the postoperative course of the BAT group with that of patients who underwent unilateral posterolateral thoracotomies (PLTs) for the same etiology.

**Results:**

Of 17 surgical procedures, there were seven BAT and 10 unilateral PLT. Mean ages at the time of the procedures were 12.9 ± 5.4 years old for BAT, and 17.4 ± 1.9 years old for PLT. Mean operative time was 173 ± 37 minutes in the BAT group, and 145 ± 39 minutes in the PLT group (*P* = 0.19). Patients received epidural analgesia in all cases; this was for a mean time of 3.8 ± 1.3 days in the BAT group, and 3.21 ± 4 days in the PLT group (*P* = 0.36). Chest tubes were removed after 3.6 ± 1.3 days in the BAT group, and 3 ± 1.2 days in the PLT group (*P* = 0.69). Total hospital stay was 7.7 ± 6.6 days in the BAT group, and 7 ± 1.2 days in the PLT group (*P* = 0.72).

**Conclusion:**

In our experience, BAT seems suitable and shows outcomes similar to those of PLT for sarcoma metastasis resection. The BAT procedure allows the manual exploration of both lungs during a single surgical intervention, and so reduces the delay of further therapies.

## Background

The general survival rate of children diagnosed with sarcoma has dramatically improved in recent decades, and now a 5-year survival rate of 80% is found for osteosarcoma [1]. However, the prognosis for patients with lung metastasis after sarcoma has remained mixed, with a 5-year survival rate varying from 30% to 40% [2,3]. Patient care consists of combined treatment with chemotherapies and multiple recurring lung resections [4].

The recommended surgical approach has been to allow a manual palpation of the pulmonary parenchyma and subsequent possible surgery [2,5-7]. Classic, two-stage thoracotomies were required to resect lung metastasis, with a variable delay of 2 to 4 weeks between the two procedures. In the longer scenario, adjuvant therapies were then postponed for a further month. For these reasons, one-stage procedures such as median sternotomy [8] or bilateral simultaneous thoracotomy [9] have been proposed, but we found no data concerning bilateral anterior thoracotomy (BAT) for this indication in the relevant pediatric literature.

The aim of our study, via the review of cases of our institution, was to determine the postoperative outcomes for children treated by using BAT for lung metastasis after sarcomas.

## Methods

The research protocol was accepted by the Ethical Committee on Human Research of the Canton de Vaud (# 212/13, May 10, 2013). Written informed consent was obtained from the patient’s guardian/parent/for the publication of this report and any accompanying images.

### Patients

Data from 2000 through 2011 were collected retrospectively. Files were reviewed for patients younger than 18 years old who had been operated on for lung metastases. In total, seven patients diagnosed with metastatic sarcoma were included in the study. Patient descriptions are shown in Table 1. All children were treated according to current protocols and received preoperative chemotherapy regardless of the type of the tumor. We then compared outcomes by using BAT against those using classic unilateral posterolateral thoracotomies (PLTs) for the same indication. All patients who required an exploration of both lungs had BAT, whereas PLT was performed only when the metastases were unilateral. No patient underwent sequential PLT for this indication, according to our team protocol.

**Table 1:** Patient descriptions

	**Age at diagnosis (year)**	**Gender**	**Histology**	**Localization**	**LM initial/relapses**	**PL thoracotomy**	**BAT**
1	1.6	M	Undifferentiated sarcoma	Hallux	Relapse	0	1
2	4.5	F	Rhabdomyosarcoma	Abdominal	Initial	0	1
3	15.4	F	Osteosarcoma	Femur	Initial	1	1
4	14.9	M	Osteosarcoma	Femur	Relapse	4	2
5	12.5	F	Osteosarcoma	Femur	Relapse	0	2
6	13.6	M	Osteosarcoma	Femur	Initial	2	0
7	9.8	M	Ewing sarcoma	Rib	Relapse	3	0

In all cases, the primary tumor was completely resected (R0). Patients with either initial or relapsed metastases were monitored, and metastasectomy was considered if metastases were not responding to chemotherapy.

### Diagnostic process and follow-up

A multiple-slice chest computed tomography (CT) scan was carried out as an initial workup, and analyzed by a senior radiologist. We compared the number of preoperative metastases with intraoperative findings. In all cases, a checkup scan was performed 3 months after the surgical procedure.

### Surgical technique

The clamshell incision required a "W" curvilinear bilateral submammary incision, extending from one midaxillary line to the opposite one in the anatomic skin and rib groove. The skin incision is carried down to the superficial pectoral fascia overlying the pectoralis major muscles, the sternum, and the fascia overlying the serratus anterior muscles at the lateral ends of the wound. The pectoralis major muscle is separated from its inferior and medial attachments and lifted up with its overlying skin and soft tissues.

The pectoral muscles are elevated to gain access to the fifth intercostal space bilaterally, whereupon the chest is entered, and the incision completed toward the sternum bilaterally. The division of the intercostal muscles continues laterally and posteriorly to maximize rib spreading. The internal mammary vessels are isolated, tied with a Vicryl 3-0 absorbable suture and sectioned. The sternum is divided transversally at the level of the fifth intercostal space with an oscillating saw.

At the end of the operation, bilateral pleural drainage is placed. The wound is closed with pericostal stitches. The sternum is repaired with two heavy metallic sutures. Muscles and subcutaneous tissues are closed in layers

### Statistics

All data are expressed as mean ± SD. We used the Student *t* test to compare the continuous variables within groups. Two-tailed *P* < 0.05 was considered statistically significant. The analysis was performed by using Prism (2013 GraphPad Software, Inc.).

## Results

### Patients

Seven BAT procedures were performed on five patients (two patients had repeated operations), with a mean age at surgery of 12.9 ± 5.4 years (Table 1). Four patients, with a mean age of 17.4 ± 1.9 years, underwent 10 unilateral thoracotomies. Two patients underwent both BAT and subsequent PLT. All patients underwent multiple wedge resections. One child in the PLT group underwent a right superior lobectomy. The mean number of nodules detected at CT scan, across both groups, was 3.47 ± 3.9 nodules per patient, and the number of resected nodules was 3.52 ± 3 nodules per patient (*P* = 0.48).

In four procedures of 17, a histologic analysis found no viable tumoral tissue and only scars. In the other patients, the metastases were still viable, despite preoperative treatments. In the BAT group, five patients of seven had bilateral nodules on CT (7 ± 6 nodules per patients), which were confirmed by the operative and histologic findings (6.6 ± 4 nodules per patient).

Concerning the two remaining patients with preoperative unilateral metastasis (one and three nodules), we decided to explore both lungs, because of their personal history of previous bilateral metastasis (and previous BAT). In one case, bilateral metastases were diagnosed and resected (one nodule on CT scan and three during the procedure) (Table 2).

**Table 2:** Surgical considerations and outcome

	**Thoracotomy**	**BAT**	
**Number of procedures**	10	7	
**Age at surgery (years)**	17.4 ± 1.9	12.9 ± 5.4	
**Operating time (minutes)**	145 ± 39	173 ± 37	NS
**Length of peridural anesthesia (hours)**	3.2 ± 1.4	3.8 ± 1.3	NS
**Chest-tube removal**	3 ± 1.2	3.6 ± 1.3	NS
**ICU stay (days)**	3.2 ± 3.5	4.6 ± 1.1	NS
**Total hospital stay (days)**	7.1 ± 1.2	7.7 ± 6.6	NS
**Time to initiation of chemotherapy (days)**	18.5	21.5	

### Surgical considerations and outcome

Mean operating time was 173 ± 37 minutes in the BAT group and 145 ± 39 minutes in the PLT group (*P* = 0.19). In all cases, patients received epidural analgesia, according to our hospital protocol, for a mean time of 3.8 ± 1.3 days in the BAT group and 3.21 ± 4 days in the PLT group (*P* = 0.36). Chest tubes were removed after 3.6 ± 1.3 days in the BAT group and 3 ± 1.2 days in the PLT group (*P* = 0.69).

Mean stay in the intensive care unit (ICU) was 4.6 ± 1.1 days in the BAT group and 3.2 ± 3.5 days in the PLT group (*P* = 0.07). Total hospital stay was 7.7 ± 6.6 days in the BAT group and 7 ± 1.2 days in the PLT group (*P* = 0.72).

No intraoperative complications were noticed; however, six postoperative complications were observed: two in the BAT group and four in the PLT group (four persistent air leaks, one pleural effusion, one lung infection; all were considered Grade 2 according to the Clavien-Dindo Classification of Surgical Complications [10]). No postoperative deaths occurred.

According to institutional protocols, postoperative chemotherapy was required after five of the lung resections. It was decided to monitor the others, as they had already followed up several regimens and/or previously experienced major complications from chemotherapy. The delays between surgical procedures and further treatment were 13, 30, and 40 days (due to postoperative pneumonia) in the BAT group and 21 days in the PLT group. No data of residual and chronic pain secondary to BAT or PLT were retrieved from the clinical data.

The mean follow-up was 5.1 ± 3.4 years. Of the seven patients treated, three survived, but two of these had relapses.

## Discussion

In the pediatric population, surgical resection of lung metastasis has been shown to improve survival in numerous malignancies, including sarcomas [2,6]. In cases of bilateral lung nodules, the classic approach was to consider sequential unilateral thoracotomies, with a variable delay of 2 to 3 weeks between procedures. Thanks to the experience of thoracic surgeons, an alternative treatment was proposed to avoid two consecutive anesthesias and the consequent risks of thoracic surgery, as well as to shorten the delay before administration of adjuvant chemotherapy, when required. This treatment included bilateral simultaneous thoracotomy or median sternotomy [8,9,11]. However, no reports exist on experiences with BAT in children or adolescents with this indication. Our present study supports BAT as a safe and useful approach for bilateral lung metastasis.

In recent decades, specialists have debated different surgical approaches to cases of bilateral lung metastasis, but no consensus has been reached. It was hypothesized that a one-stage approach could lead to better outcomes, as it avoids a second procedure (including anesthetic risks) and reduces the delay before administration of adjuvant chemotherapy. This one-stage approach has been reported in the pediatric population [8,9,11].

**Figure 1:**
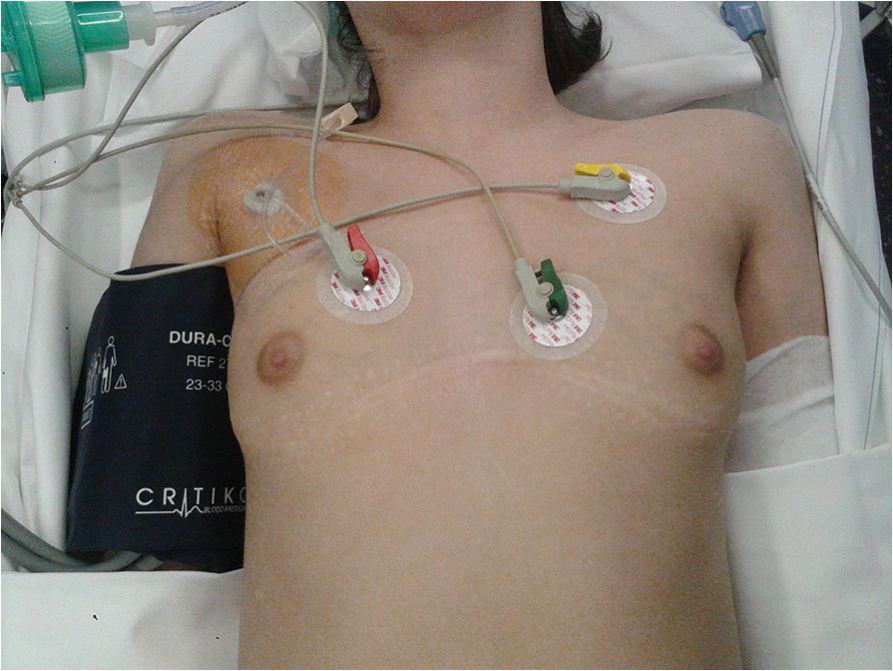
A 15 year old girl who underwent two bilateral anterior thoracotomy for metastasis resection: appreciate the curvilinear bilateral submammary incision.

Median sternotomy was initially described for adults with this indication. The limitations of this approach were the suboptimal exposure of the left lower lobe and the postoperative recovery. In pediatric cases, mobilization of the left lower lobe often results in hemodynamic or ventilation problems, as Fuchs *et al.* described [11]. However, conversely, Tsai *et al*. [8] reported a better exploration of the left lower lobe, without recurrence of metastasis in this area. Our study revealed that BAT offers good access to both lungs, avoids certain physiopathologic inconveniences, and does not require a change of the patient’s position during surgery, as it does during bilateral simultaneous PLT. The wide access to pulmonary parenchymas on both lungs during this procedure permits the accurate inspection and palpation that is essential in open surgery, including the upper lobes. We decided to perform a PLT for the patient who required an upper lobectomy, because only one metastasis was discovered on the preoperative workup. Nevertheless, BAT allows a perfect access to the upper lobes but was not indicated in this particular case. As with median sternotomy, we found that the operating time was 30 min longer than in unilateral PLT, but both sides were explored in the same procedure.The postoperative course showed that complication rates, the amount of painkillers needed, and the time with a chest tube in place were all similar. Patients were discharged within 7 days of surgery regardless of the surgical approach, with a real trend toward a reduced hospital stay in both groups. Moreover, the esthetic outcome was also good (Figure 1), even in the cases with multiple BAT (two cases), and no sternal disruption was noticed.

Minimally invasive surgical approaches are uniformly considered not indicated in a curative goal [12], and should be reserved for classic biopsy, diagnosis, and staging. Gossot *et al.*[13] recently described criteria to determine which patients could benefit from thoracoscopy rather than an open approach to treating sarcoma relapses. They concluded that this controversial approach should be considered in cases of “two or fewer nodules, and subpleural nodules that are suitable for a thoracoscopic resection, which means that free margins can be obtained”. This was based on their experience with adults, but as yet, no confirmed data exist for drawing these conclusions in pediatric patients. Promising results were recently obtained with lung nodules localized by CT scan before thoracoscopic resection [14]. However, the absence of indications for mini-invasive approaches for sarcomas in children has been suggested by the results of a French multicenter study [5].

Classic management of open surgery focused on improving survival rates by resecting all metastases. This was based on the discrepancy between the number of metastases detected by CT scan and the number of palpated and thus resected ones [7,15]. This difference has become scarcer in more recent reports, probably because of the increased precision of thoracic CT scans. Although it is too early to reconsider the use of thoracoscopy as part of the therapeutic management possibilities for lung metastasis in children, further investigations and clinical trials must estimate whether this technique has a future.

Our study has some limitations. First, it has the usual drawbacks of retrospective studies of rare pathologies in children. Our experience is limited (seven clamshell incisions), and the groups are rather heterogeneous. However, all procedures were performed by the same experienced surgical team, whose know-how in pediatric surgical oncology had defined its management for the last 10 years in our instititution.

Second, the group monitored may be a concern. Because of the decision to perform BAT for this indication, we have no experience of simultaneous bilateral or sequential PLT, or of sternotomy. We are therefore not able to discuss precisely the different technical aspects of the other procedures.

Third, the main concern about BAT is potential morbidity. Our study found no more complications than in the PLT group monitored, even in repeated thoracotomies. Furthermore, pain management was exactly the same across the study, including epidurals for all patients when the pleural tubes were placed and then oral analgesia on their removal. In our study, the patients operated on with BAT did not experience more pain than the PLT group, according to the duration and level of required painkillers. None of the patients required oxygen supply to aid with lung function after surgery, even after the removal of the epidural catheter. No reported clinical data on exercise lung function was found in the literature concerning children. As previously described [9], we wanted to highlight that BAT is a suitable approach for treating bilateral metastasis in children.

## Conclusion

From our study, we conclude that bilateral anterior sternothoracotomy (clamshell incision) is a suitable option for the management of lung sarcoma metastasis in children and adolescents. It allows a single operation to explore both lungs accurately, resect secondary lesions, and potentially reduce delays in the administration of adjuvant chemotherapy when required. Moreover, the postoperative courses were similar to those for classic posterolateral thoracotomies.

## Competing interest

The authors declare that they have no competing interests.

## Authors’ contributions

AO manuscript writing. RG data collection and manuscript writing. KP manuscript revision. JMJ design and supervision of the study, manuscript revision. All authors read and approved the final manuscript.
